# Inference and interrogation of a coregulatory network in the context of lipid accumulation in *Yarrowia lipolytica*

**DOI:** 10.1038/s41540-017-0024-1

**Published:** 2017-08-11

**Authors:** Pauline Trébulle, Jean-Marc Nicaud, Christophe Leplat, Mohamed Elati

**Affiliations:** 1 Micalis Institute, INRA, AgroParisTech, Université Paris-Saclay, 78350 Jouy-en-Josas, France; 2 Université d’Évry, Évry, 91000 France; 3 CNRS-UMR8030/Laboratoire iSSB, Évry, 91000 France; 4 CEA, DRF, IG, Genoscope, Évry, 91000 France; 5 Université Paris-Saclay, Évry, 91000 France

## Abstract

Complex phenotypes, such as lipid accumulation, result from cooperativity between regulators and the integration of multiscale information. However, the elucidation of such regulatory programs by experimental approaches may be challenging, particularly in context-specific conditions. In particular, we know very little about the regulators of lipid accumulation in the oleaginous yeast of industrial interest *Yarrowia lipolytica*. This lack of knowledge limits the development of this yeast as an industrial platform, due to the time-consuming and costly laboratory efforts required to design strains with the desired phenotypes. In this study, we aimed to identify context-specific regulators and mechanisms, to guide explorations of the regulation of lipid accumulation in *Y. lipolytica*. Using gene regulatory network inference, and considering the expression of 6539 genes over 26 time points from GSE35447 for biolipid production and a list of 151 transcription factors, we reconstructed a gene regulatory network comprising 111 transcription factors, 4451 target genes and 17048 regulatory interactions (YL-GRN-1) supported by evidence of protein–protein interactions. This study, based on network interrogation and wet laboratory validation (a) highlights the relevance of our proposed measure, the transcription factors influence, for identifying phases corresponding to changes in physiological state without prior knowledge (b) suggests new potential regulators and drivers of lipid accumulation and (c) experimentally validates the impact of six of the nine regulators identified on lipid accumulation, with variations in lipid content from +43.2% to −31.2% on glucose or glycerol.

## System biology: gene regulatory network for lipid production

Bioinformatics allow us to get new insight in yeast gene regulation for the biological production of lipid-derived industrial compounds. This work, led by Jean-Marc Nicaud and Mohamed Elati from the INRA and University of Evry, aimed to identify the gene regulatory network and new regulatory elements associated with lipid production in *Yarrowia lipolytica*, a yeast of industrial interest. Using context-specific gene expression data and a data mining approach, the team was able to build a model of gene regulation from which they identified the main regulators as well as the cooperative relationships between them. These predictions were then confirmed experimentally. Additionally, the team managed to determine different stages of lipid production using a new measure of regulator activity: the influence. These tools will be useful to model and design industrial yeast for various applications.

## Introduction

*Yarrowia lipolytica* is a non-pathogenic dimorphic ascomycetous yeast that has been used by scientists for fundamental and applied studies^1, 2^ and for its utility as an industrial platform for the production of lipid-derived compounds.^3–6^ Indeed, *Y. lipolytica* can grow in hydrophobic environments, using complex hydrocarbons, hydrophobic substrates (e.g., n-alkanes, fatty acids) and cheap industrial by-products as substrates.^7^ This species has also been engineered to extend the variety of substrates it can use, and it can now grow on biomass products, such as cellobiose and raw starch.^8, 9^ Metabolically, this yeast tends to store lipids under conditions of nitrogen limitation, an adaptation favoring survival in the face of nutrient deficiency developed during the course of evolution and providing interesting possibilities for use as an industrial platform. Several potential uses of this yeast have been considered, but its metabolism has been studied principally for its potential to produce various compounds through fatty-acid metabolism, including lipids, unusual fatty acids, aromas, dicarboxylic acid or TCA-cycle intermediates, such as succinic acid and 2-ketoglutaric acid.^4, 10–13^ A broad range of tools has also been developed and validated for efficient genetic engineering in *Y. lipolytica*.^14–17^ Safety assessments have been carried out, and this species has been classified as generally regarded as safe of use (GRAS),^18^ making it ideal for use in industrial biotechnology.^3, 19^ However, we currently know very little about the regulators involved in lipid accumulation of *Y. lipolytica*. This lack of knowledge is limiting the development of this yeast as a metabolic engineering platform, as it remains time-consuming and costly to develop strains with the desired phenotype. Gene regulatory networks (GRNs) can be seen as the interface through which genotype-environment interactions give rise to the phenotype. Indeed, GRNs act like the “operating system” of the cell, adjusting its behavior to external conditions and causing changes in the amounts of transcripts, protein concentrations and metabolic fluxes, through the actions of effector molecules, such as transcription factors (TFs) or other proteins (e.g., phosphatases and kinases involved in post-transcriptional modifications). Regulatory networks are therefore of great importance, to provide insight into the adaptive behavior of living systems in a condition-specific manner whilst making it possible to predict the state of the cell and its responses to environmental constraints.

However, the systematic characterization of GRNs is not always straightforward, as little is known about most of these networks, and they are often highly interconnected. The existing research tools for regulatory network reconstruction^20, 21^ and interrogation^22^ have greatly contributed to our understanding of biological systems. Such networks were especially obtained for well known model organism such as *Saccharomyces cerevisiae*.^23–26^ The difficulty lies in the growing gap between high-throughput biological data production and the mathematical models and analytical tools used to derive a systems context from the data. These networks are usually reverse engineered from large-scale transcriptomic samples and evidence of physical interactions (ARACNE,^27^ WGCNA,^28^ GENIE3,^29^ LICORN^30^). Our reverse engineering approach, Hybrid- learning co-operative regulation networks (h-LICORN),^30, 31^ combine a data mining technique and a numerical linear regression to effectively infer GRN (see Materials and Methods) and is original principally in terms of the incorporation into the model of the cooperativity between co-regulators, rendering it more relevant for the comprehension of complex phenotype that are likely to be regulated by several regulators rather than by a single one, as shown by us and others in the yeast *S. cerevisiae*,^30, 32^ as well as in human.^31, 33^

In this work, we aimed to identify regulators and transcriptional programs associated with lipid accumulation, to improve our understanding of this process and to identify candidate regulators able to alter the phenotype of this yeast. We inferred a network from transcriptomic data during lipid accumulation and interrogated it, to highlight context-specific regulation and for the experimental validation of some of the candidates identified. One key breakthrough in the exploration of these networks was the shift of focus from the expression of regulators to their influence, through evaluations of the expression of target genes,^33, 34^ with the aim of detecting master regulators.

## Results

### Coregulatory network assembly in the context of lipid accumulation

We reconstructed a coregulatory network from our GSE35447 transcriptomic data set, deposited in NCBI Gene Expression Omnibus database^35^ (https://www.ncbi.nlm.nih.gov/geo/query/acc.cgi?acc=GSE35447). These data were generated with the Agilent platform (A-GEOD-15177—Agilent-031148 *Yarrowia lipolytica* V2) and correspond to 80 samples taken during a time-course experiment in which Carbon/Nitrogen (C/N) ratio was increased to induce nitrogen-limiting conditions and lipid accumulation. Lipid yield and content are dependent on the nature of nutrient limitation. N limitation is the most widely used to induce lipid production, as it gives the best conversion yield with glucose.^36^ Samples were obtained from a D-stat culture, where the dilution rate was kept constant while one of the cultivation parameter (temperature, C/N ratio) was modulated at a constant rate),^37^ at 26 different time points, for three biological replicates. The data for 6539 genes were normalized (see Materials and Methods) then processed by CoRegNet (Bioconductor package) to produce a genome-wide regulatory network. Briefly, CoRegNet is a workflow that use the h-LICORN algorithm^31^ to mine candidates GRNs set of co-activators and co-inhibitors for each genes. Various types of evidence, such as protein–protein interactions (PPI), can then be incorporated to support cooperative interactions into a score of validated interactions. Candidates GRNs are then evaluated on their ability to describe the gene expression data and their evidence score. Once the best GRN had been selected, a cooperative network is reconstructed, based on the shared TF targets, making it possible to identify coregulatory relationships solely on the basis of the gene expression data provided. We improved the reliability of the inferred network by running CoRegNet with a minCoRegSupport parameter of 0.2 and a curated list of 151 TFs identified by our team from previous studies, homology and sequence analyses. PPI for *Y. lipolytica* were downloaded from the STRING database,^38^ which provide interactions based on either experimentation, homology with better known organism such as *S. cerevisae*, or prediction. These evidences were therefore incorporated into the network (*P*-value = 3.12e-42). The resulting network (**YL-GRN-1**) contains 111 transcription factors, 4451 target genes and 17,048 regulatory interactions. Further information about network inference is available in Materials and Methods. The association between gene name and official common name is provided in Supplementary Table 1.

### TF activity over nitrogen limitation highlights specific patterns during lipid accumulation

From YL-GRN-1, sample-specific TF activity can be estimated through its targets expression. We proposed a measure, the TF influence, to assess its activity. This measure is based on a Welch’*t*-test between the expression of the activated and repressed targets genes in a given samples (more details in Materials and Methods). TF influence was shown robust to noise^33^ and can be used to decrease the dimensionality of the data, thereby facilitating the visualization of patterns through an integrative view accessible in the CoRegNet package. TF influence was calculated for replicate means, to obtain a single value for each of the 26 time-point that was representative of the variability between the three technical replicates. The TF influence heatmap generated in this way provides a visual representation of transcriptional programs.

**Fig. 1:**
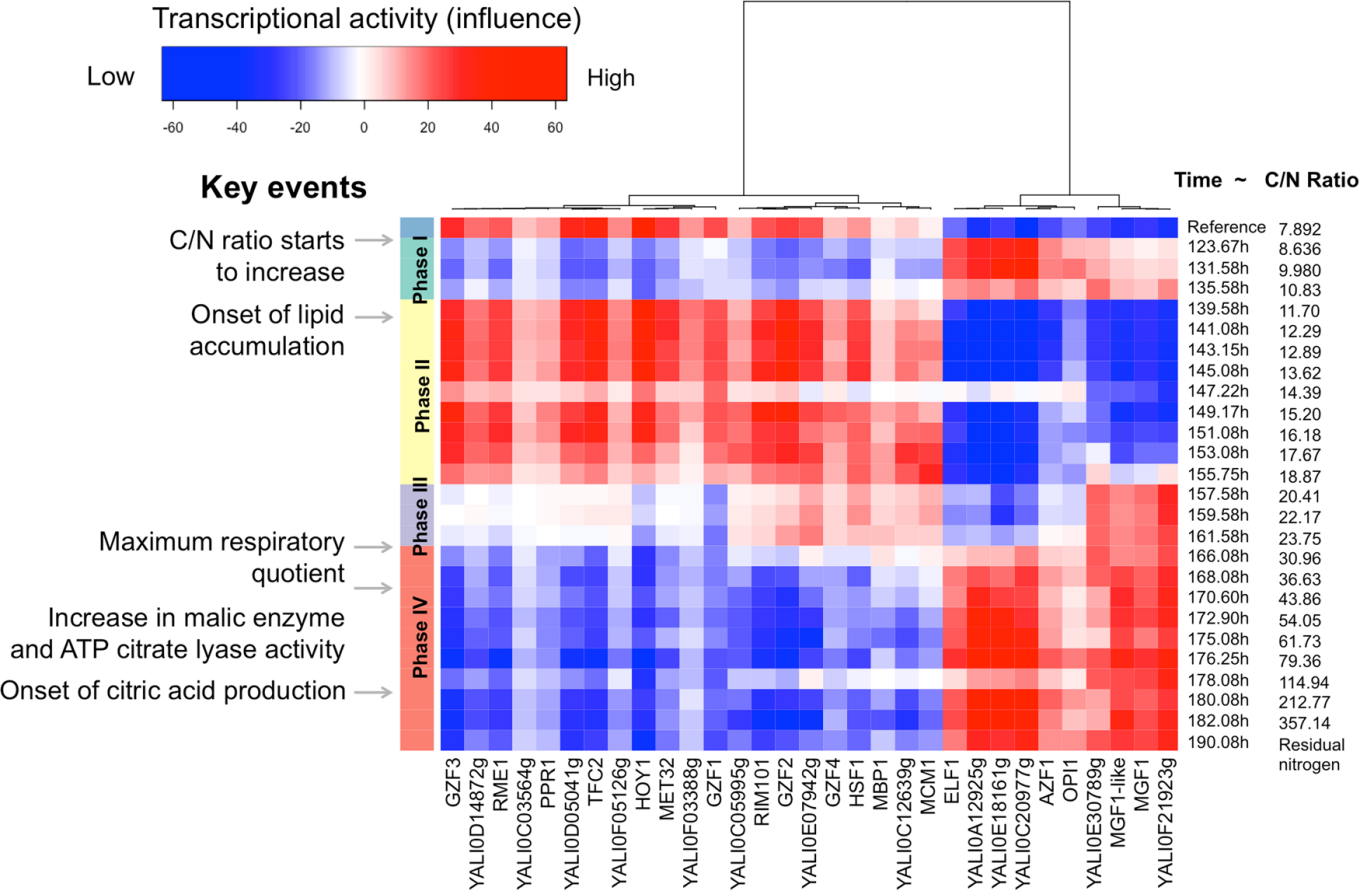
Heatmap of TF influence as a function of C/N ratio during a time-course experiment. Four main phases were identified on the basis of changes in influence pattern: phase I (*t* ±  = 123.67 h, C/N ratio = 7.89), phase II (*t* = 139.58 h, C/N ratio = 8.63), phase III (*t* = 157.58 h, C/N ratio = 20.41), and phase IV (*t* = 166.08 h, C/N ratio = 30.96). These phases are shown on the *left* in *turquoise, yellow, purple and red*, respectively. Negative and positive influences are indicated from *blue to red*, with color intensity proportional to the influence value. Time and C/N ratio are indicated on the *right*, as described by Ochoa-Estopier and Guillouet ^37^ and in the GSE35447 data set

Patterns were identified in the transcriptional program, defining several phases during the GSE35447 D-stat experiment (Fig. 1). Neither carbon nor nitrogen was limiting in the reference state (C/N ratio = 7.89), but four other phases could be defined, as follows: (a) Phase I (*t* ±  = 123.67 h, C/N ratio = 8.63) corresponds to the early response to decreasing nitrogen levels. This pattern was first observed at about *t* = 120 h, when nitrogen became limiting .^37^ This phase persisted until the C/N ratio reached 11.70. Below this value, nitrogen limitation triggered new regulators, leading to lipid accumulation in the second phase. (b) Phase II (*t* = 139.58 h, C/N ratio = 11.70) appeared to be associated with early adaptation to nitrogen limitation: at this stage, yeast metabolism adapts to the nitrogen limitation of the environment, so as to maintain maximal growth while performing the normal functions, despite resource limitation. This phase immediately preceded the onset of lipid accumulation, which was first detected at about 140 h. (c) During phase III (*t* = 157.58 h, C/N ratio = 20.41), many regulatory changes were observed that could be seen as a remodeling of the regulatory network to adapt from short-term nitrogen limitation to long-term nitrogen limitation. Finally, (d) phase IV (*t* = 166.08 h, C/N ratio = 30.96) corresponded to long-term adaptation to nitrogen depletion. The changes in TF influence pattern correlated with the experimental observations reported in a previous study,^37^ not only at 120 h and 140 h, but also at 165 h, which coincides with the time at which respiratory quotient and lipid accumulation reach their peak values. The experimental observations associated with lipid accumulation were therefore consistent with the estimated activity of the TFs considered here. Some TFs seemed to lose their influence or to be activated before others, suggesting a hierarchy of the response to nitrogen limitation and identifying particular TFs as potential drivers of the transition between physiological phases. For example, *YALI0E30789g*, *MGF1-*like *(YALI0B19602g)*, *MGF1* and *YALI0F21923g* were activated during phase III, whereas other TFs were not activated until phase IV.

### Identifying the most influential TFs in lipid accumulation and the master regulators of lipid-associated genes

We evaluated the importance of each TF throughout the whole experiment and the different phases, by ranking TF according to their influence, with the RobustRankAggreg R package .^39^ For each phase, TF influence was computed and ranked from positive to negative value as we considers that the regulator is active only when it activates its set of activated genes (*A* ^*r*^) and represses its set of repressed genes (*I* ^*r*^), as expected by the network reference model which is reflected by a positive Welch *t*-test value while a negative value represent the “absence” of TF activity with the repressed genes (*I* ^*r*^) more expressed than the activated genes (*A* ^*r*^). The regulator is more active when this value is higher. However, the ranking of the TF over the whole experiment was carried out using the absolute value of the TF influence to assess the impact of the TF in every phase over both their *A* ^*r*^ and *I* ^*r*^. The full rankings are shown in Supplementary Table 2. The top 10 most influential TFs over the whole experiment were *YALI0C20977g*, *RME1*, *YALI0E18161g*, *GZF3*, *GZF2*, *TFC2*, *YALI0F21923g*, *HOY1*, *MGF1*-like and *RIM101*. These TFs had the strongest influence over the entire experiment, but they were not active in the same phase Mixed patterns were also observed in phase III, with some TFs displaying changes in their influence earlier than others (e.g. *MGF1*, *YALI0E30789g*, *YALI0F21923g*) **(**Fig. 1).

**Fig. 2:**
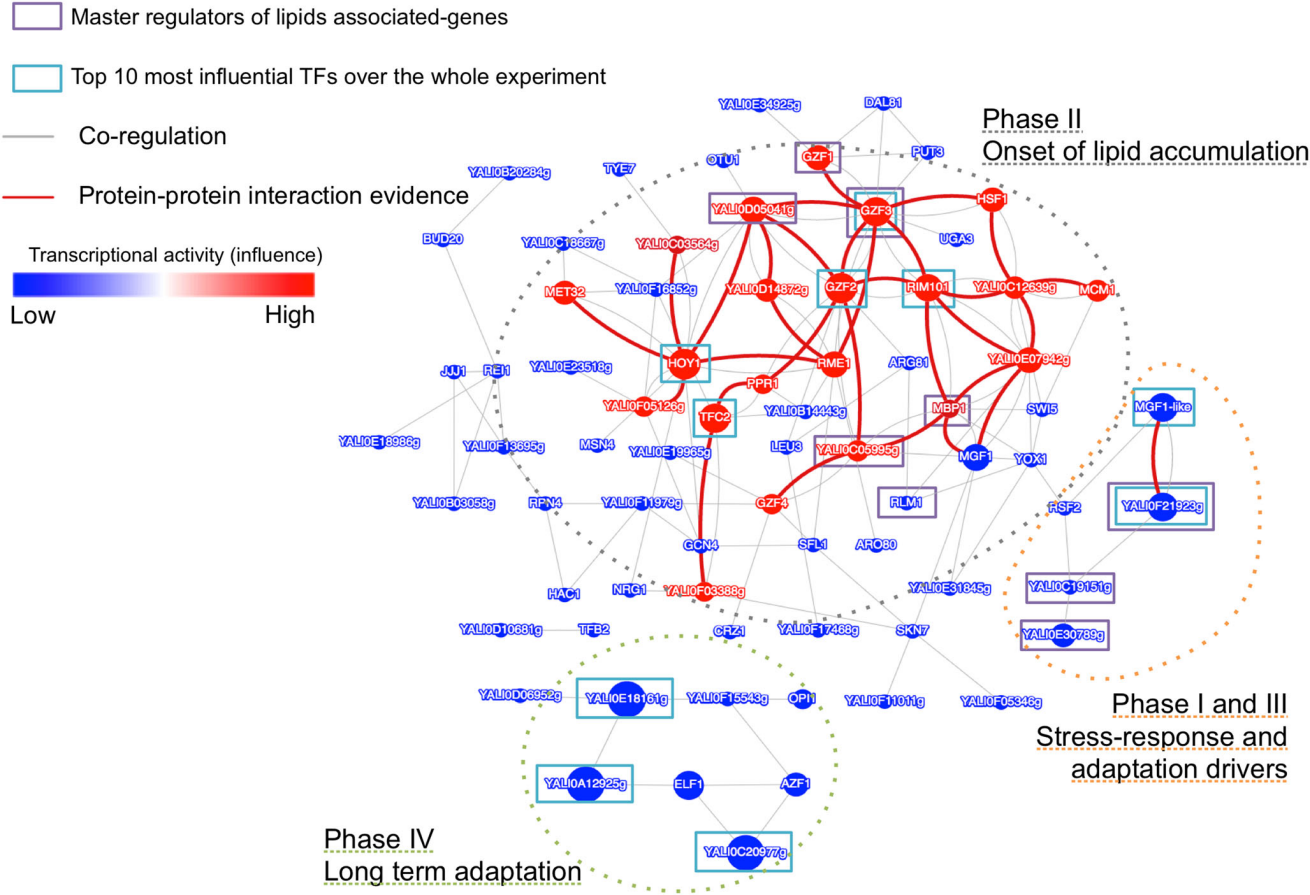
Heterarchy**—**Cooperativity network for *Yarrowia lipolytica* (YL-CoRegNet-1) constructed from YL-GRN-1, which was inferred from our transcriptomic data set under nitrogen limitation, GSE35447. Nodes represent transcription factors (TFs), whereas *gray edges* indicate co-regulatory relationships. *Red edges* are co-regulatory relationships for which evidence of protein–protein interactions has been obtained. Node size and color represent the influence of the corresponding TFs during the onset of lipid accumulation (phase II). *Red* indicates a positive influence whereas *blue* indicates a negative influence. Color intensity and node size are proportional to the influence value

We retrieved a list of 282 *Y. lipolytica* genes from the Panther webserver^40^ on the basis of their association with GO slim biological processes relating to lipids (lipid transport, phospholipid metabolism, lipid metabolism processes, or protein lipidation. See Supplementary Table 3). From this list, we identified master regulators on the basis of YL-GRN-1 (Table 1). The projection of both the top 10 most influential TFs and master regulators over the YL-CoRegNet-1 cooperativity network (Fig. 2) highlighted the high degree of connectivity of these TFs within a portion of the network and suggested that they acted in synergy during lipid accumulation.

**Table 1:** Master regulators for lipid-associated genes in *Y. lipolytica* as retrieved from the Panther webserver on the basis of GO slim BP

Master regulators of lipid-associated genes and their *P*-values	
*YALI0F01562g*	4.519e-05
*GZF1*	5.552e-04
*YALI0E30789g*	0.0025
*MBP1*	0.0098
*YALI0D05041g*	0.0107
*RLM1*	0.0124
*YALI0F21923g*	0.0137
*YALI0C19151g*	0.0169
*YALI0C05995g*	0.0442
*GZF3*	0.0477

### Validation of TF activity as a tool for identifying physiological phases

A second network, YL-GRN-2, corresponding to the transition from biomass production to lipid accumulation, was reconstructed from our previous transcriptomic studies (GSE29046)^41^ consisting of 11 sampling points, regularly spaced over the period of fed-batch culture, to validate the potential of TF influence for identifying relevant time points corresponding to important physiological changes in the absence of prior knowledge. The data set was studied with the following CoRegNet parameters: minCoregSupport = 0.25, minGeneSupport = 0.2. The influence heatmap for YL-GRN-2 presented three clear phases corresponding to the stages in the transition from biomass production to lipid accumulation identified and relating to (A) biomass production, (B) early lipid accumulation and (C) late lipid accumulation, respectively (see Supplementary Fig. 2).

### Use of a cooperativity network to identify evidence-supported coregulatory relationships and to identify new candidate co-regulators

A co-regulation network (YL-CoRegNet-1) was reconstructed from YL-GRN-1, as shown in Fig. 2. In this network, each node represents a TF, and the gray edges correspond to co-regulation by two regulators with a sufficient number of target genes in common. In particular, the red edges represent co-regulation for which evidence of protein–protein interactions has been obtained. Evidence-supported co-regulatory relationships are well represented in the network and are highly interconnected. A review of the similarity-based annotations associated with the recovered TFs available from GRYC (http://gryc.inra.fr), genolevures, NCBI and from previous studies^41, 42^ highlighted the presence of TFs known or assumed to be involved in lipid metabolism (e.g., *GZF1*, *GZF2* or *GZF3*), carbon or nitrogen metabolism (e.g., *AZF1*, *YALI0F01562g*, *YALI0D14872g*, *NRG1*, *YALI0C19151g*, *CAT8*) and growth or hyphal formation (e.g., *RME1*, *HOY1*, *REI1*, *MGF1*, *MGF1*-like), and of several TFs displaying no similarity or known functions (e.g*., YALI0F15543g*, *YALI0E18161g*, *YALI0F15543g)*. Some of the less common, but nevertheless interesting, functions of the TFs were associated with amino-acid metabolism, which is known to be affected by lipid accumulation .^43^ For instance, *GCN4* is associated with amino-acid metabolism generally, whereas *LEU3* is specifically associated with leucine, *PUT3* is associated with proline and *ARG81* is associated with arginine. Some of these TFs were identified as co-regulators with others TFs with similar functions, such as *GZF2*, *GZF3* and *GZF1*, *GZF4* all of which encode GATA-binding zinc finger proteins, but others act as co-regulators with non-trivial TFs, generating new hypotheses for further investigations of the regulation of lipid accumulation. Several modules were manually identified by projecting TF influence from the different phases onto the cooperativity network thanks to the interactive visualization interface from CoRegNet. Those sets of TFs activated in each phase were highly interconnected with one another into region of high density in the network (Supplementary Fig. 1). The largest module corresponds to the TFs associated with phase II, as shown in Fig. 2. Two other modules can be identified, corresponding to the TFs activated during phases I and III, and those activated during phases I and IV.

### Construction of overexpression mutants for experimental validation of the impact of the most influential TFs on lipid accumulation profile

**Fig. 3:**
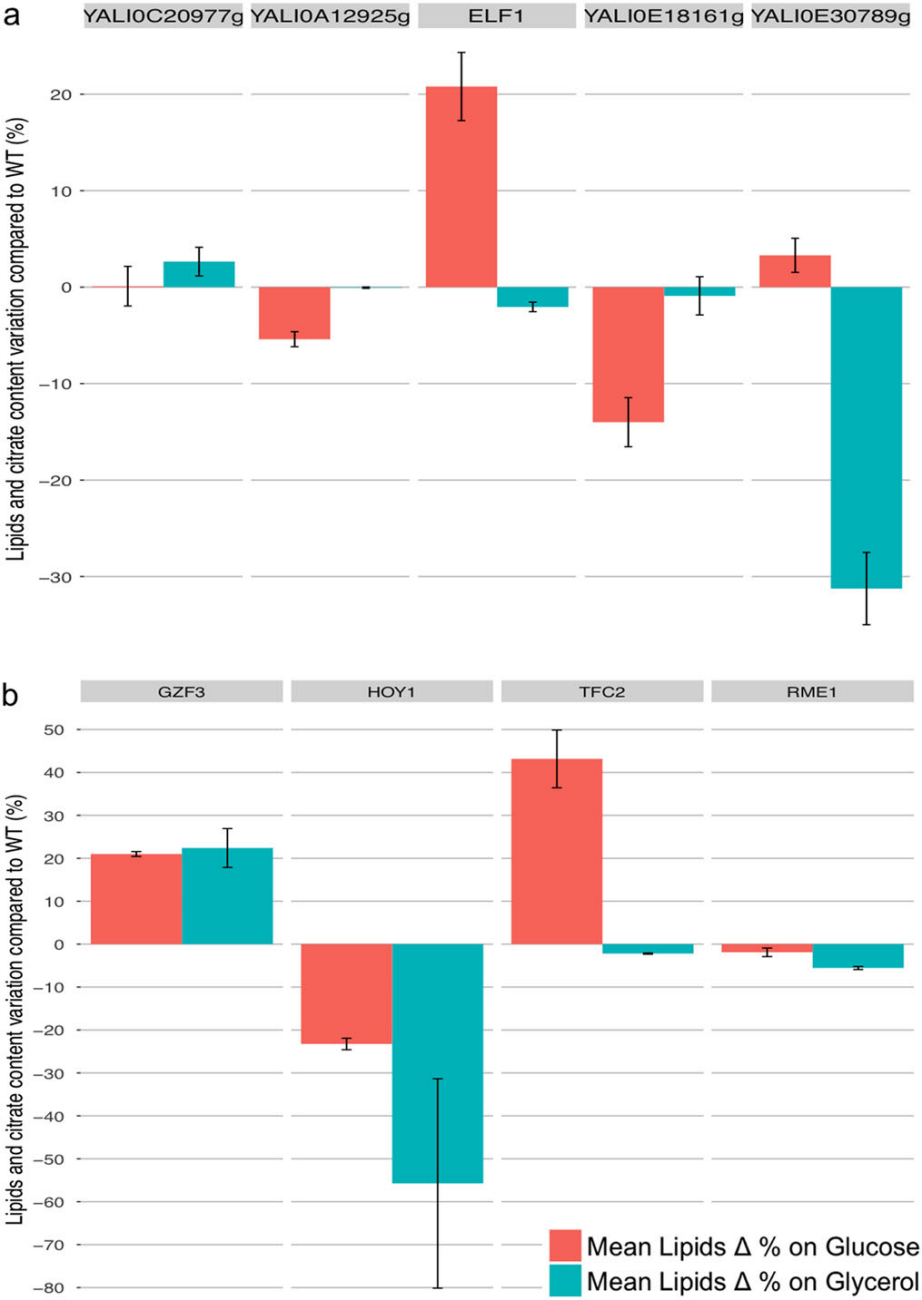
Mean percentage differences in lipid accumulation profile of overexpressing TFs mutant relative to the wild type with their s.d. Differences were considered significant if there was a change of at least ± 10%. TF-overexpressing strains were selected on the basis of their ranks during phase I (**a**) and phase II (**b**)

To confirm the impact of the identified TFs in triggering lipid accumulation, TFs were individually overexpressed in the *Y. lipolytica* wild-type strain JMY2810, with the Gateway systematic overexpression system developed in our laboratory (17, Leplat C., Rossignol T. *et coll*., unpublished), as described in the materials and methods. Lipid content was assessed after 72 h of culture in minimal medium, with either glucose or glycerol as the carbon source and ammonium as the nitrogen source, with a C/N ratio of 3. Lipid content was determined by gas chromatography. We report here the effects on lipid content of the five most influential TFs during phases I and II, based on YL-GRN-1. The effects of the most influential TFs during phase I, *YALI0C20977g*, *YALI0A12925g*, *ELF1*, *YALI0E18161g* and *YALI0E30789g*, are described in Fig. 3a. We were unable to obtain a strain overexpressing *GZF2*. The effects of the four most influential TFs during phase II, *GZF3*, *HOY1*, *TFC2* and *RME1*, are shown in Fig. 3b.

Three of the nine overexpression strains had an improved lipid content on glucose, 43.2% and 20.8% higher than the wild type for *TFC2* and *ELF1*, respectively. *GZF3* and *HOY1* overexpression led to altered phenotypes on both glucose and glycerol, with *GZF3* overexpression resulting in 21.0% higher levels of accumulation on glucose and 22.4% higher levels on glycerol, whereas *HOY1* overexpression resulted in much lower lipid contents on glucose (23.3% lower) and glycerol (55.7% lower). The lipid contents of the strains overexpressing *YALI0E18161g* and *YALI0E30789g* were decreased in a medium specific-manner, with a 14.0% decrease on glucose and a 31.2% decrease on glycerol, respectively. Finally, three of the overexpression strains, those for *YALI0C20977g*, *RME1* and *YALI0A12925g* (*RME1*-like), displayed no significant modification of lipid content.

## Discussion

On the basis of the inferred cooperativity network and our proposed measure of influence, several regulators were highlighted as co-regulators in the context of lipid accumulation in *Yarrowia lipolytica*. Multiple pathways and functions are represented in the network, in particular, regulators of growth (e.g, *TFB2*, *AZF1*, *MGF1*), filamentation (e.g, *HOY1*, *SFL1*), nitrogen utilization (e.g, *GTZ1* to 4) and genes regulating amino-acids biosynthesis, such as *ARO80*, *ARG81*, *MET32*, *GCN4*, or *LEU3*, acting as coregulators during the different phase identified. Indeed, the projection of influence onto the network for each phase (Supplementary Fig. 1) helps with studying the temporality of the regulation and the presence of coregulators densely connected into «modules» sharing the same influence pattern.

As seen during phase I, *AZF1, OPI1, YALI0C20977, YALI0E18161g, YALI0A12925g* are among the TFs activated during the first phase. These TFs are activated just after the C/N ratio starts to increase and are assumed to be associated with the first response to nitrogen depletion, with an alteration of growth and cell cycle regulation, and may provide a regulatory pulse enabling the yeast to deal with nitrogen limitation by redirecting carbon towards lipid accumulation and entering phase II. While *AZF1* and *OPI1* are known to be associated with growth and repression of phospholipid synthesis respectively, only few is known about the three others regulators, however, GO term enrichment of *YALI0E18161g* repressed targets revealed an over-representation of genes associated with cell cycle (4.19E-02).

TFs activated during the second phase of biolipid accumulation gather various functions and form the biggest «module» and as well as the denser part of the cooperativity network.

At this stage, all the 4 GATA-zinc finger TFs (*GZF1, GZF2, GZF3, GZF4*) presents in the network are active with *GTZ2* and *GTZ3* being the more co-regulated. The presence of those regulators during this phase is consistent with recent validation of their involvement in the regulation of nitrogen metabolism in *Y. lipolytica* ^44^ but further analysis of the network and shared target between *GZF1* and *GZF3* also suggest an over-representation of genes related to fatty-acid metabolic process (2.68E-02), while *GZF1* is considered as a master regulator for both lipid and amino-acid associated genes and *GZF2* is co-regulators of both *ARG81* and *LEU3*. Those observations are supporting their potential role in lipid regulation, as well as the imbrication of nitrogen utilization and amino-acids pathways for the regulation of lipid accumulation.

Among the influential TFs during phase II, *HOY1* and *TFC2*, two coregulators, seem to have a less direct effect on lipid accumulation, as they are involved in filamentation and transcription initiation. When overexpressed, *HOY1* decreases lipid accumulation, probably due to its role in yeast-to-hyphae transition. When growing, the yeast form requires the mobilization of lipids for membrane synthesis. Thus, even if the yeast accumulates more lipids, they are immediately remobilized, decreasing lipid content. The activation of this TF at the onset of lipid accumulation may thus coincide with post-transcriptional alterations or the action of a co-regulator. Indeed, a second regulator could be able to make use of the new lipids generated under the influence of *HOY1*, but might interfere with the remobilization of lipids, shifting the balance towards lipid accumulation. Candidate regulators for this role include *RME1*, a repressor of meiosis, for which there is strong evidence for a role as a co-regulator of *HOY1* but whose overexpression has no specific effect on the accumulation phenotype despite being shown to be among the most influential TFs during phase II. However, it also worth to note that *HOY1* included amino-acid related TFs (*MET32*), as well as TFs for which no function are known among its co-regulators, which may also be candidates of interests (e.g, *YALI0C03584g*). As in phase I, a TF module activated before the shift toward citric acid production could provide a regulatory pulse toward this pathway. In particular, the set of TFs activated during phase III and IV includes a large number of master regulators of the 267 genes with GO-slim BPs relating to amino acids (*P*-value <0.05) including *YALI0F21923g* and *YALI0E30789G*, whose roles are unknown*, YALI0C19151g*, a CAT8-like TF likely to be involved in growth and non-fermentative growth conditions, and *MGF1*-like, a growth factor, which may be potential drivers of the long-term adaptation to nitrogen depletion in phase IV. In addition, it worth to note that those same regulators seems to regulate significantly beta-oxidation among their predicted activated targets (*P*-value 7.04E-07, 5.64E-06, 4.84E-02 for *YALI0F21923g*, *YALI0E30789g* and *YALI0C19151g*, respectively). Activation of beta-oxidation during long-term adaptation may be explained by the use of lipids degradation as a source of energy in the context of long-term nutrient depletion, resulting in citric acid production as by-product as well.

TFs were ranked on the basis of their influence. This approach decreased the number of dimensions, but it cannot necessarily be concluded that the TFs not retained with this approach are not involved in lipid accumulation. It is also important to note that not all influential TFs belong to the list of lipid master regulators. This difference between the lists of master regulators and most influential TFs may reflect the involvement in lipid accumulation of mechanisms affecting not only lipid pathways, but also the metabolism of the entire cell, which is consistent with previous observations^41, 43, 44^ and support the hypothesis that lipid accumulation is a consequence of change in carbon fluxes rather than an enhanced lipid metabolism. In addition, several regulators shown to be differentially expressed during lipid accumulation^41, 43^ were retrieved in our network as coregulators (e.g, *GZF3*, *GZF2*, *ARG81, YALI0C19151g, TFB2*) while others were found to have non-trivial partners for which functions are yet to be found. The most influential TFs may not necessarily have the most direct effects on the lipid pathway. Instead, their influence might reflect their final overall effect and their ability to have a significant effect on various pathways in nitrogen-limiting conditions, indirectly promoting lipid accumulation. Consistent with this, five of the nine significant amino-acid master regulators were among the most influential TFs (Supplementary Fig. 3).

## Conclusion

Lipid accumulation in the oleaginous yeast *Y. lipolytica* is a process of considerable industrial interest for the environment-friendly production of high-value compounds derived from lipids, such as biofuels, bioplastics and other biomolecules with properties of interest. However, metabolism results from complex interplay between the environment, genetic background and regulation, with cells adopting various states and presenting different phenotypes. An understanding of the role of gene regulatory networks in lipid accumulation is therefore of key importance for both the design of improved strains and to increase our knowledge of this yeast species. We inferred a genome-scale regulatory network, YL-GRN1, consisting of a total of 111 TFs acting as co-regulators of target genes during lipid accumulation under nitrogen limitation. The influence of the TFs was estimated in the different samples and a matrix of influence over time and increasing C/N ratio was generated.

Changes in influence over the course of the experiment were consistent with the observed physiological changes and stages of lipid accumulation. Indeed, the sensitivity of *Y. lipolytica* to nitrogen limitation led to changes in TF influence patterns at each key time point. The influence matrix is therefore a powerful tool for highlighting physiological changes in the absence of prior knowledge. From this matrix and the YL-CoRegNet-1 cooperativity network, we were able to identify different modules providing potential drivers of the lipid accumulation phases and possible co-regulators of interest. Finally, TFs were ranked and the TFs with the highest ranks during phases I and II were overexpressed in a wild-type strain, with the Gateway overexpression system. Six of the nine mutants obtained presented altered phenotypes, with lipid contents differing from that of the wild type by more than 10%, validating our approach to the identification of context-specific TFs.

Future studies should focus on computational developments (a) to improve our ability to combine the proposed co-regulatory model with genome-scale metabolic models^45^ (b) to select the most informative combination of TF knockout strains and environmental conditions based on the integrated regulatory network.^46^ Moreover, understanding regulatory processes is a key element in the development of synthetic biology with the aim of designing and engineering large, self-adaptive, coupled regulatory and metabolic systems at whole-genome scale for useful purposes, such as the production of valuable compound.^47^

## Materials and Methods

### Experimental setting and transcriptomic data collection

Chemostat and D-Stat experiments were performed in a 3 L stirred tank bioreactor with a working volume of 1.5 L, with a Braun Biotech International Biostat B (Sartorius AG, Germany) and MFCS/win 2.0 acquisition software. The temperature was regulated at 28°C and the pH at 5.6 by the online addition of 5 M NaOH. Continuous culture was initiated 11 h after inoculation, when the glucose consumption was complete. For chemostat culture, the bioreactor was fed continuously with mineral medium (devoid of (NH_4_)_2_SO_4_) supplemented with 23 g L^−1^ glucose at 0.108 L h^−1^. The bioreactor was fed with a second reservoir containing 60 g L^−1^ (NH_4_)_2_SO_4_ at 0.0117 L h^−1^, corresponding to a C/N ratio of 7.75 molC.Nmol^−1^. The working dilution rate was 0.08 h^−1^. The feed rate of the mineral medium supplemented with glucose was kept constant at 0.120 L h^−1^, whereas that for (NH_4_)_2_SO_4_ followed a smooth linear decrease, from 0.0117 L h^−1^ to 0.0003 L h^−1^ for 50 h, corresponding to an increase in the C/N ratio from 7.75 to 357,14 molC.N mol^−1^. All other parameters were kept constant. For more details on the experimental setting, see Ochoa-Estopier et al.^37^

Frozen samples were treated by mechanical disruption, with a bead beater (Microdismembrator, Braun, Germany) and a tungsten bead (Ø ~ 7 mm), for 2 min at 2600 r.p.m. The resulting cell powder was recovered and further processed for RNA purification with the RNeasy Midi Kit (Qiagen, The Netherlands), according to the manufacturer’s instructions. Samples were treated for labeling with the Low-Input Quick Amp labeling kit (Agilent, USA), according to the manufacturer’s protocol, and hybridization was performed according to Agilent’s general protocol. Scanning was performed with an Agilent scanner and images were further processed with Feature Extraction v10.0 (Agilent, USA).

Data were processed and normalized with the Limma Bioconductor package .^48^ Local background estimates were corrected by the “normexp + offset” method, using an offset value of 10. A scale normalization method was applied to normalize background between arrays. ID REF = VALUE = log2(fluorescence), based on background-subtracted, normalized data. The processed data are publicly available from the NCBI GEO data repository under the name **GSE35447**. The resulting data set corresponds to 80 samples for 6539 genes, with three technical replicates of 26 time-points with an increasing C/N ratio, plus four replicates of the reference point.

### Constructing TF-target Gene regulatory network (YL-GRN-1) and TF-TF cooperativity network (YL-CoRegNet-1)

Complex phenotypes are believed to arise from cooperative transcriptional programs rather than from regulation by a single regulator. CoRegNet was developed to study such programs and to reconstruct large-scale context-specific co-regulatory network from transcriptomic data. It was shown to outperform other network inference algorithms, particularly for small sample numbers,^31^ an advantage when studying a non-conventional yeast, such as *Y. lipolytica*, for which few transcriptomic datasets are available.

CoRegNet uses an algorithm, h-LICORN (hybrid-Learning Cooperative Regulation Network), to infer a list of GRNs from a discretized transcriptomic data set and a list of known regulators on the basis of a frequent itemset mining approach.^30, 31^ Briefly, in a first step, it efficiently searches the discretized gene expression matrix for sets of co-activators and co-repressors by frequent items search techniques and locally select combinations of co-repressors and co-activators as candidate subnetworks. In a second step, it determines for each gene the best sets among those candidates by running a regression. h-LICORN was shown to be suitable for cooperative regulation detection [5,6].

The continuous data can be used alone to refine the original network by selecting for each gene the GRN with the best $\bar R$ ^2^ score based on the linear model used to estimate the expression. However, CoRegNet can also refine GRNs by incorporating evidence into the network using an integrative selection algorithm proposed by the modENCODE consortium^49^ and applies it to the selection of local GRN models. In essence, the goal is to score each GRN (each interaction in the original method) using both the transcriptomic data and the integrated evidences to select the set of best GRN. Each GRN is scored by the inference method h- LICORN and by each of the integrated data set. Finally, GRN are given the proportion of validated interactions as a score. Following this, to each GRN is associated as many scores as they are integrated regulatory and cooperative datasets in addition to the network inference $\bar R$ ^2^ score, all which range from 0 to 1. The original study proposes two approaches to merge the scores, an unsupervised and a supervised approach. While both are implemented in the CoRegNet package, the unsupervised approach was shown by the authors to have better performances. It is simply an unweighted average of each of the scores. Finally, for each gene, the GRN with the maximum merged score is selected. The refined network obtained is then transformed into a cooperativity network, based on the common targets of regulators.

We identified regulators and regulatory states associated with lipid accumulation in *Y. lipolytica*, by applying CoRegNet to the preprocessed **GSE35447**, as described above. CoRegNet was run with a default minCoregSupport = 0.1, with a curated list of 151 TFs retrieved from previous publications and from homology analysis. *Y. lipolytica* interactome data relying on either experimentation, in-silico prediction, or most commonly on homology analysis were downloaded from the STRING database,^38^ and used as evidence for network refinement.

CoRegNet is freely available as a Bioconductor package.

### Sample-specific TF activity estimation

We used the transcriptomic data and the highest-ranked GRN to compute a sample-specific value of influence for each TF with a sufficient number of targets. This approach models the h-Licorn inferred GRN structure by comparing for each regulator *r* the distribution of its activated A^r^ and repressed I^r^ genes (∀r ∈ V^R^, targets(r) = (A^r^, I^r^)). This model is based on the work in^33^ where the influence measure was introduced to estimate the activity of a regulator through a Welch t-test by comparing the distribution of the expression of A^r^ and I^r^. The influence of a regulator *r* is computed as follows: $\frac{{\overline {E\left( {{A^r}} \right)} - \overline {E\left( {{I^r}} \right)} }}{{\sqrt {\frac{{{\mu }_{{A_r}}^2}}{{\left| {{A_r}} \right|}} + \frac{{{\mu }_{{I_r}}^2}}{{\left| {{I_r}} \right|}}} }}$ where $E\left( {{A^r}} \right)$ and $E\left( {{I^r}} \right)$ are respectively the set of expressions of the activated and repressed genes in the samples. $\overline {E\left( {{A^r}} \right)}$and $\overline {E\left( {{I^r}} \right)}$ are their respective means and ${\mu }_{A_r}^2$ and ${\mu }_{I_r}^2$ are their s.d. The most influential TFs in a specific set of conditions are associated with large differences in expression between repressed and activated targets, and are represented as larger nodes in the network. Similarly, the TF influence value can be projected onto the network and incorporated into an integrative heatmap-based visualization. The influence of each TF in each sample is represented by colors of different intensities: red indicates a positive influence, implying stronger expression of activated genes than of repressed genes, whereas blue indicates a negative influence, with the opposite pattern. The more intense the color, the greater is the influence of the TF. The robustness of this measurement was assessed, for each TF, by correlation analysis, using the original network and a partially permuted version of the network with increasing levels of noise. Similar tests were performed, analyzing the correlation of TF influence on subparts of the network validated by regulatory evidence. In all comparisons, influence was significantly more robust and consistent with the validated network .^33^ This measurement estimates TF activity, which cannot be determined by experimental approaches. The default parameter minTarget = 10 was used to calculate influence.

### Context-specific transcriptional program visualization

Both the network and its influence heatmap can be visualized through a dedicated tool implemented in CoRegNet, using Shiny application, with features for displaying the main sets of co-regulators in specific samples, stages or subtypes. The network is represented as a graph, in which each node is a regulator, each gray edge is a co-regulatory relationship and each colored edge is a co-regulatory relationship for which evidence is provided. The size and color of the nodes are proportional to the differential expression and value of TF influence, respectively.

### Experimental validation

Mutants were constructed by inserting the TF expression cassette (*URA3*ex-pTEF-TF) into JMY2566 (*MAT*a, *ura3*::pTEF-RedStar2-*LEU2ex*-Zeta, *leu2-270*, *xpr2-322*, Ura-, Leu + ) as described by Leplat et al.^17^ The wild-type strain JMY2810 (*MAT*a, *ura3*::pTEF-RedStar2-*LEU2ex*-Zeta-*URA3*ex-pTEF, *leu2-270*, *xpr2-322*, Ura + , Leu + ) was used as the wild-type control. Cassettes containing the TF gene of interest were overexpressed under the control of the constitutive pTEF promoter from the *TEF1* gene, which encodes translation elongation factor-1α. Yeasts were grown in YNB medium with either 3% glucose or glycerol and a C/N ratio = 30 for 72 h at 28°C. Lipid content was determined by gas chromatography. Lipid content duplicates were averaged, standard deviations were plotted, and the results were expressed as a percentage variation between the control strain JMY2810 and TF-overexpressing mutants. (Leplat C., Rossignol T, unpublished).

### Panther webserver

Panther webserver tools^40^ were used to retrieve genes associated with GO terms related to lipids and amino-acids as well as for gene ontology enrichment using *Yarrowia lipolytica* all genes as reference set and default setting in addition to Bonferroni correction.

### Data availability

All data and tools mentionned in this article are freely accessible, in particular, transcriptomic data that support the findings of this study have been deposited in NCBI Gene Expression Omnibus database with the accession code GSE35447 (https://www.ncbi.nlm.nih.gov/geo/query/acc.cgi?acc=GSE35447). CoRegNet is freely available as a Bioconductor package.

## Electronic supplementary material

Supplementary Figures

Supplementary Table 1

Supplementary Table 2

Supplementary Table 3

Supplementary Table 4
