# Supplementary material for: Inference and interrogation of a coregulatory network in the context of lipid accumulation in Yarrowia lipolytica
Source: NPJ Syst Biol Appl. 2017 Aug 11;3:21. doi: 10.1038/s41540-017-0024-1 (PMC5554221; doi:10.1038/s41540-017-0024-1)
Supplement: Supplementary file 1 — Supplementary Figures [file 41540_2017_24_MOESM1_ESM.docx]

*Supplementary Figure 1: Network YL-CoRegNet-1 and TF influence at the different phases
Supplementary Figure 2: Influence heatmap and network* GSE29046

*Supplementary Figure 3:* M*aster regulators of genes associated with amino-acid GO terms (pvalue <0.05)*

*Supplementary Table 1: Transcription factors genes names and common names
Supplementary Table 2: TFs ranking according to the different phases*

*Supplementary Table 3: Genes list associated with lipids and amino acids retrieved form Panther*

*Supplementary Table 4: Co-regulators from the network*

Supplementary Figure 1 Co-regulatory network of *Yarrowia lipolytica* inferred from a transcriptomic dataset under nitrogen limitation. Nodes represent transcription factor (TF) while grey edge represent a co-regulatory relationship. Red edges are co-regulatory relationship for which evidences were provided. The node size and colors represent the influence of the corresponding TFs during the onset of lipids accumulation. Red is associated with a positive influence whereas blue is related to a negative influence.

**
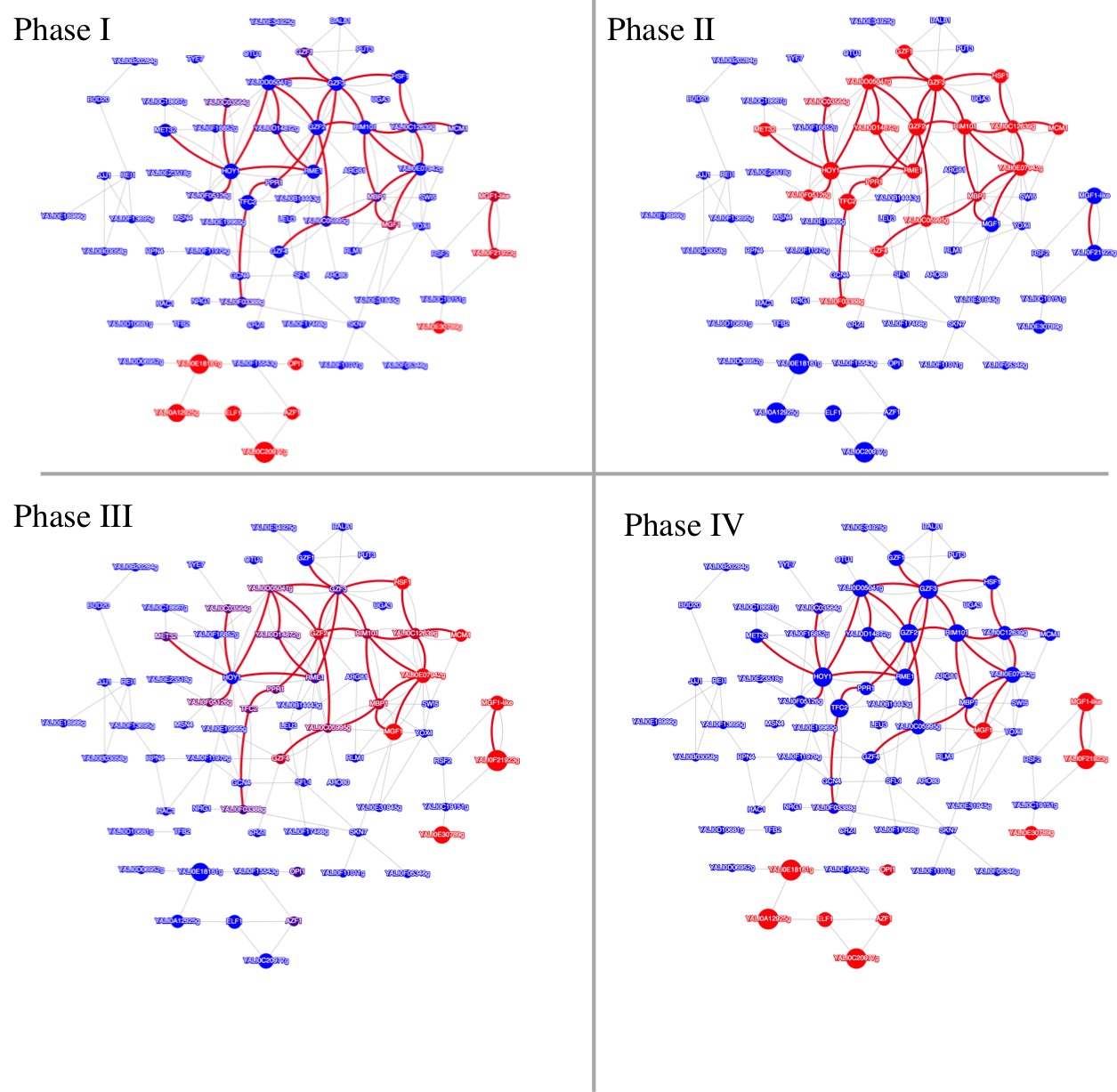
**

**Supplementary Figure 2** YL-Coreg-2 cooperativity network and influence heatmap based on the GRN reconstructed from GSE29046 (YL-GRN-2)


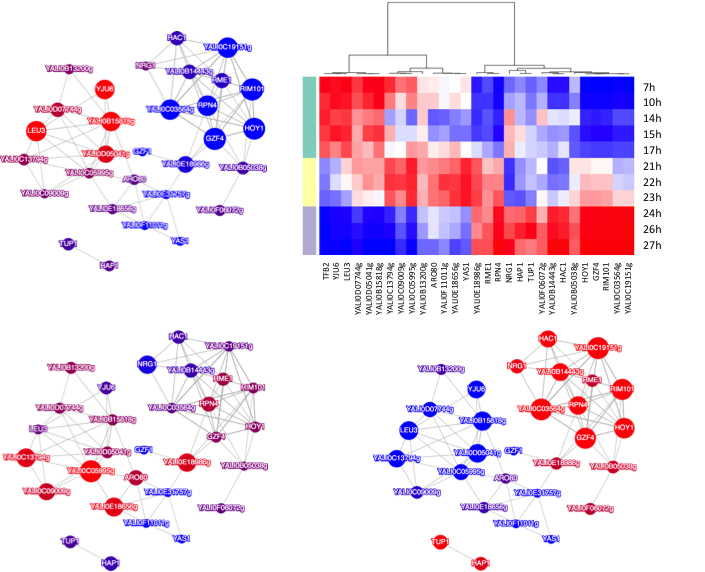


**Supplementary Figure 3** Master Regulators of amino-acid associated genes

| Master regulators | YALI0E07942g | GZF1 | YALI0E34925g | MGF1 | YALI0C19151g | YALI0F21923g | MGF1-like | RSF2 | MBP1 |
| --- | --- | --- | --- | --- | --- | --- | --- | --- | --- |
| P-values | 0.00017 | 0.00135 | 0.00226 | 0.00334 | 0.00350 | 0.00552 | 0.00777 | 0.02719 | 0.04211 |
